# Live-cell imaging of subcellular structures for quantitative evaluation of pluripotent stem cells

**DOI:** 10.1038/s41598-018-37779-x

**Published:** 2019-02-11

**Authors:** Ken Nishimura, Hiroshi Ishiwata, Yuta Sakuragi, Yohei Hayashi, Aya Fukuda, Koji Hisatake

**Affiliations:** 1 Laboratory of Gene Regulation, Faculty of Medicine, University of Tsukuba, 1-1-1 Tennodai, Tsukuba, Ibaraki, 305-8575 Japan; 2 Optical Technology R&D Department 2, Optical System Development Division, Olympus Corporation, 67-4 Takakura-machi, Hachioji, Tokyo, 192-0033 Japan; 3 iPS Cell Advanced Characterization and Development Team, Bioresource Research Center, RIKEN, 3-1-1 Koyadai, Tsukuba, Ibaraki, 305-0074 Japan

## Abstract

Pluripotent stem cells (PSCs) have various degrees of pluripotency, which necessitates selection of PSCs with high pluripotency before their application to regenerative medicine. However, the quality control processes for PSCs are costly and time-consuming, and it is essential to develop inexpensive and less laborious selection methods for translation of PSCs into clinical applications. Here we developed an imaging system, termed Phase Distribution (PD) imaging system, which visualizes subcellular structures quantitatively in unstained and unlabeled cells. The PD image and its derived PD index reflected the mitochondrial content, enabling quantitative evaluation of the degrees of somatic cell reprogramming and PSC differentiation. Moreover, the PD index allowed unbiased grouping of PSC colonies into those with high or low pluripotency without the aid of invasive methods. Finally, the PD imaging system produced three-dimensional images of PSC colonies, providing further criteria to evaluate pluripotency of PSCs. Thus, the PD imaging system may be utilized for screening of live PSCs with potentially high pluripotency prior to more rigorous quality control processes.

## Introduction

Pluripotent stem cells (PSCs), including embryonic stem cells (ESCs) and induced pluripotent stem cells (iPSCs), have variations in their capacity to differentiate^1^. This variability is caused by genetic and epigenetic differences that arise during derivation, induction, and subsequent maintenance of PSCs^2,3^. The variation of pluripotency in PSCs may potentially compromise the utility of PSCs in biomedical researches and their applications in regenerative medicine. For example, PSCs with low pluripotency may generate a population of somatic cells that could be contaminated with undifferentiated or partially differentiated cells, which pose a risk of tumor formation or low efficacy after transplantation^4,5^. Therefore, selection of PSCs with high pluripotency is essential to ensure the safety and efficacy of PSC-derived cells. The selection, however, requires standardized procedures, which include morphological observation, surface marker analysis, whole genome sequencing, genome-wide expression profiling, *in vitro* differentiation and teratoma formation. Such rigorous procedures for quality control are costly and time-consuming, necessitating development of fast and inexpensive screening of live PSCs with high pluripotency prior to the rigorous quality control procedures.

Traditionally, selection of live PSCs with high pluripotency utilizes imaging methods that require fluorescent labeling of cells by immunostaining or gene transfection^6,7^. Such invasive methods, however, may be inadequate for clinical applications in regenerative medicine because of inevitable damage or loss of observed cells. To circumvent this, more recent studies reported label-free and non-invasive approaches, some of which are combined with computational data processing, to evaluate pluripotency of PSCs^8–10^. These methods typically utilize the morphological features of cells and colonies but not of subcellular structures due to the limited resolving power of microscopy. Because subcellular structures also undergo massive morphological changes in response to reprogramming, assessing the structural changes at the subcellular level could be equally informative for evaluating the degree of pluripotency.

One of the subcellular structures that are altered dramatically during reprogramming is mitochondria. Mitochondria are few and small in ESCs^11,12^, which originate from the inner cell mass where oxygen is low^13^ and glycolysis is the main source of energy production^14^. By contrast, mitochondria are numerous and large in differentiated somatic cells, which depend more on oxidative phosphorylation for efficient energy production^15^. As a consequence, reprogramming somatic cells into iPSCs is accompanied by a metabolic shift from oxidative phosphorylation to glycolysis, concomitant with changes in structure and function of mitochondria^16,17^. Indeed, iPSCs that are reprogrammed to different degrees show an inverse relationship between their pluripotency and mitochondrial activities^18^. Thus, if observed in a non-invasive manner, morphological changes of subcellular structures such as mitochondria may serve as a useful marker to evaluate the pluripotency of PSCs.

Non-invasive visualization of subcellular structures has been enabled by recent development of differential interference contrast (DIC) microscope combined with retardation modulation^19,20^ and two switchable orthogonal shear directions^21–23^ such as an orientation-independent differential interference contrast (OI-DIC) microscopy^24–28^. These microscopes allow quantitative measurement of subcellular structures, providing information about not only morphology but also the density and dynamics of subcellular structures. We also reported a similar technique termed retardation modulated differential interference contrast (RM-DIC) microscopy, which allows three-dimensional (3D) measurement of the microstructures of phase objects^29–32^.

Here we developed an improved RM-DIC system, termed PD imaging system, which processes and integrates two orthogonal RM-DIC images into a single image. Like OI-DIC microscopy and others, the PD imaging system captures quantitative information from biological samples without cell staining or labeling to visualize subcellular structures inside a live cell. The visualized subcellular structures could be quantified to distinguish the degrees of pluripotency among PSC colonies as well as different regions within a single colony. The 3D structure of a PSC colony, reconstructed by the PD imaging system, was found to serve as a predictive indicator of pluripotency. Thus, the PD imaging system may contribute to establish a simple and quantitative method to select for high-quality PSCs without any staining or labeling of cells.

## Results

### An improved RM-DIC imaging system permits visualization of subcellular structures

We previously developed an RM-DIC imaging system, which extracts phase components from a DIC image using three images with different retardations (±θ, 0)^30,31^. The extracted phase components are used for reconstructing a two-dimensional (2D) phase image of an object, termed as an RM-DIC image (Supplementary Fig. S1). In addition, multiple RM-DIC images along the z-axis may be vertically stacked to produce a 3D image of phase components^32^. Both types of these images visualize morphological features of an object, especially of its surface microstructures^30–32^.

**Figure 1:**
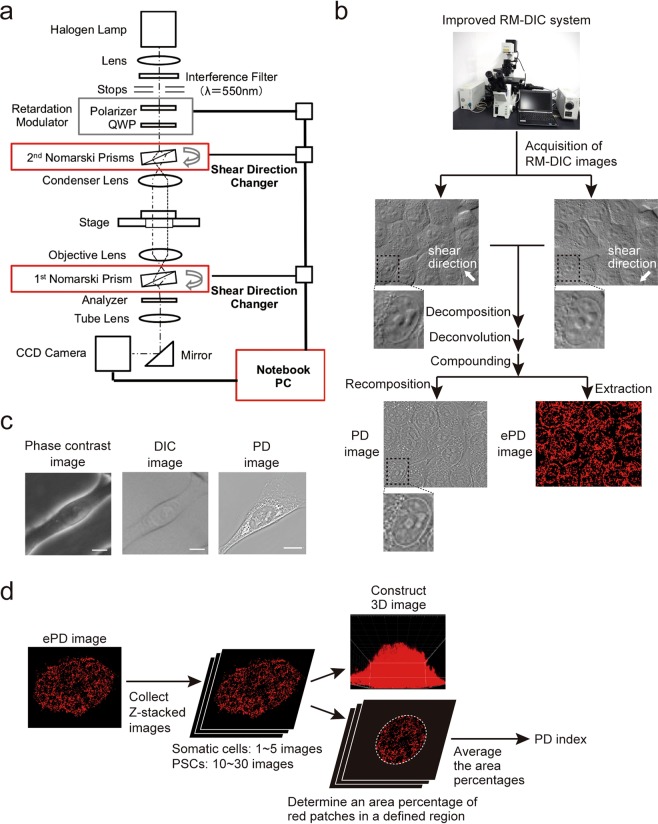
PD imaging system. (**a**) Block diagram of the PD imaging system. Red boxes indicate devices added to the conventional RM-DIC system. Abbreviations: QWP, quarter wave plate; CCD, charge-coupled device. (**b**) Scheme of producing a PD image and an ePD image using the PD imaging system. The insets in three images (two RM-DIC images and a PD image) correspond to the same region containing a nucleus. A more detailed process of producing a PD image from the two RM-DIC images is described in Supplementary Fig. S2. (**c**) Images of NIH3T3 cells observed under three different types of microscopy. Scale bars, 10 µm. (**d**) Scheme of producing 3D ePD images and derivation of the PD index.

To enable the RM-DIC imaging system to more clearly observe subcellular structures inside live cells, we equipped the RM-DIC system with two shear direction changers to obtain two orthogonal images, similar to the previously reported microscopes^24^. The shear direction changers are controlled by a PC (Fig. 1a, *red*) and rotate two prisms mechanically to acquire RM-DIC images along two orthogonal directions (Fig. 1b)^33^. Each RM-DIC image was decomposed into three components (background, refraction, and structure components) to remove the background component (Supplementary Fig. S2, *Step 1*). Then, the refraction and structure components were deconvoluted to obtain refraction and structure phases, respectively (Supplementary Fig. S2, *Step 2*). The refraction and structure phases from two RM-DIC images were compounded into compound refraction and structure phases, respectively (Supplementary Fig. S2, *Step 3*), which were then recomposed into a single image (Supplementary Fig. S2, *Step 4*). The obtained image was independent of shear direction and allowed clearer visualization of subcellular structures (Fig. 1b). For example, nuclear membrane, visible only partially in each RM-DIC image with a single shear direction, becomes visualized in its entirety in the recomposed image (Fig. 1b). We named the new system as Phase Distribution imaging system (PD imaging system) and its derived image as a PD image. Compared with either a conventional phase-contrast microscope or DIC microscope, this new system produces a more detailed image of subcellular structures (Fig. 1c). Although the use of mechanical shear direction changers renders image acquisition somewhat slow (~3 seconds per 6 images), the PD imaging system allows quantifiable visualization of subcellular structures, in a similar manner to OI-DIC^24^.

To quantify the subcellular structures, we utilized the compound structure phase, which contains the highest spatial frequency derived from fine structures in live cells (Supplementary Fig. S2)^33^. In this study, we focused on the granular spots in cytoplasm, which were observed clearly in both PD images (Fig. 1b) and compound structure phase images (Supplementary Fig. S2). We extracted the compound structure phase from its middle range, which roughly corresponded to the granular spots, by setting lower and upper thresholds (Supplementary Fig. S3a,b). These thresholds largely excluded the compound structure phases of nucleoli and cell membranes as well as that of intensely white regions, which probably consist of lipid droplets and apoptotic cells (Supplementary Fig. S3). The extracted compound structure phase was then represented in red-based pseudo-color to produce a binary image, termed an extracted PD image (ePD image), in which red patches mostly represent granular spots in cytoplasm. (Figs 1b and S2, *Step 5*). We also quantified the total area of red patches in whole cells or a whole colony. As cells and colonies are thicker than each ePD image, we collected ePD images with 2-µm pitch along the z-axis of cells (up to 5 sections) or colonies (up to 30 sections) and produced a 3D ePD image (Fig. 1d). We then averaged area percentages of the red patches in the serial images that span cells or a colony along the z-axis to define the PD index (Fig. 1d).

### The ePD image and PD index reflect the mitochondrial content

**Figure 2:**
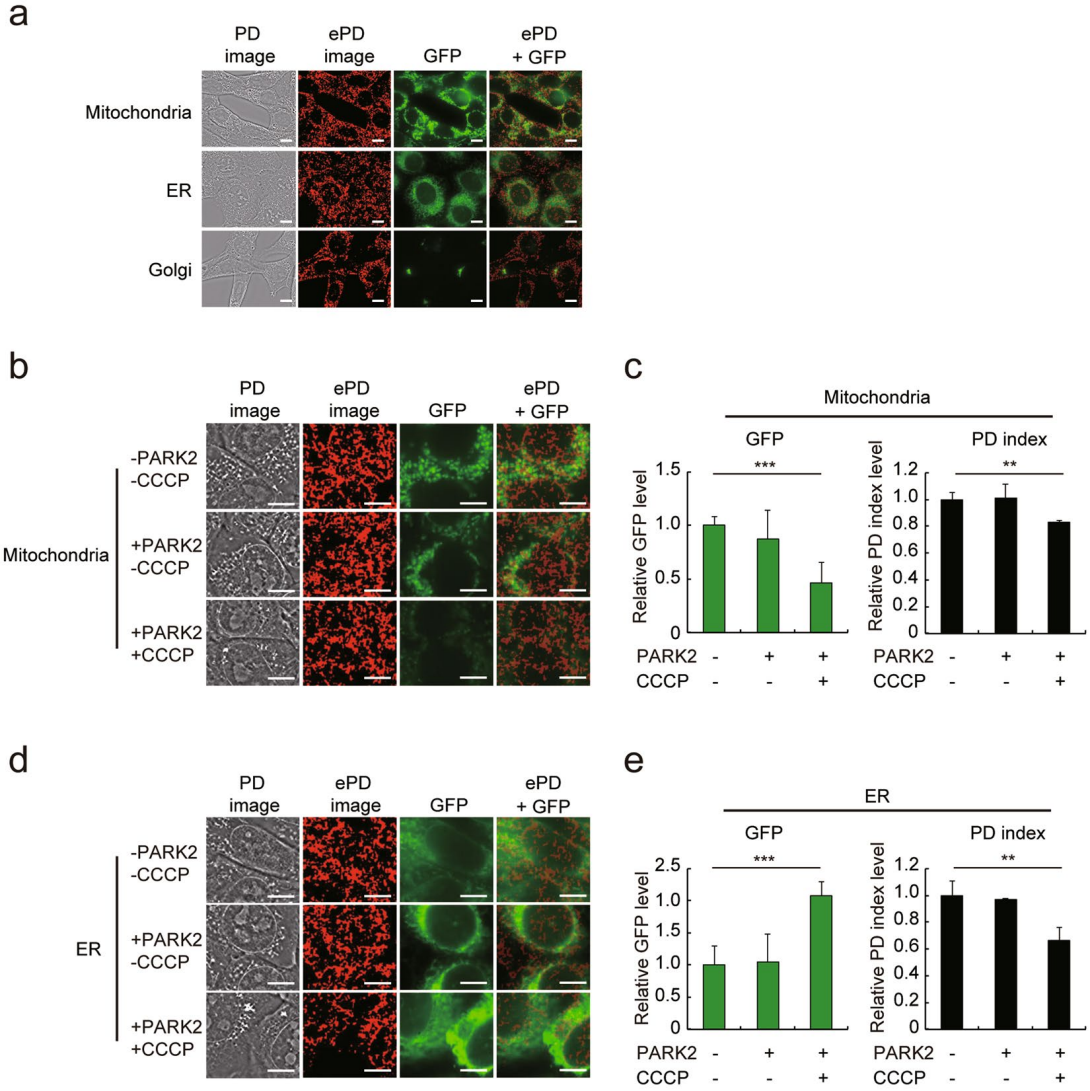
Assessment of subcellular organelles by the ePD image and PD index. (**a**) Overlapping localization of the red patches with mitochondria, the endoplasmic reticulum (ER) or the Golgi apparatus in ePD images. GFP fused with a localization signal of mitochondria, the ER or the Golgi apparatus was stably expressed in NIH3T3 cells, and the cells were observed by the PD imaging system to acquire PD images and GFP fluorescence images. Mitochondria, ER, and Golgi on the left side of images indicate localization of the expressed GFP. (**b**,**c**) Relationship of the mitochondrial content to the ePD image and PD index. NIH3T3 cells expressing GFP with a mitochondria-localization signal were induced to undergo mitophagy by expression of *Park2* with or without treatment in the presence of 10 µM CCCP for 48 h. The PD imaging system was used to acquire PD images and GFP fluorescence images as well as to produce ePD images (**b**). The PD index and the level of GFP fluorescence were quantified from corresponding images (**c**). (**d**,**e**) Relationship of the ER content to the ePD image and PD index. NIH3T3 cells expressing GFP with ER-localization signal were analyzed in essentially the same way as in (**b**,**c**). Data represent means ± SEM of at least five images. **P < 0.01, ***P < 0.005. Scale bars, 10 µm.

The RM-DIC and the PD imaging system produce images because of distinct phase amounts of an object. As lipid bilayer has a relatively high refractive index within a cell and thereby generates a distinct phase amount, the PD image obtained from RM-DIC images principally visualizes membranous organelles. Indeed, PD images of NIH3T3 cells revealed the nucleus as well as granular spots in cytoplasm, which appeared to co-localize with mitochondria, the endoplasmic reticulum (ER), or the Golgi apparatus (Fig. 2a). Extraction of the middle range of the compound structure phase, which was used to produce ePD images, de-emphasized the nuclear and cell membranes, while mostly capturing granular spots in cytoplasm as red patches (Figs 2a and S3). To identify which structure produces the red patches in ePD images, we transfected NIH3T3 cells with a plasmid expressing modified green fluorescent protein (GFP) fused with a localization signal to mitochondria, the ER, or the Golgi apparatus^34–36^ (Fig. 2a). As is evident from minimum overlap between the red patches and the fluorescence of Golgi-localized GFP (Fig. 2a, *bottom panels*), the Golgi apparatus does not represent the red patches in cytoplasm. However, the relative contribution of mitochondria (Fig. 2a, *top panels*) and the ER (Fig. 2a, *middle panels*) to the red patches was not well defined from the merged images.

To better define the relative contribution of mitochondria and the ER to the red patches in the ePD image, we reduced the mitochondrial content by expression of *Park2* and addition of carbonyl cyanide m-chlorophenylhydrazone (CCCP), a combination of which strongly induces mitophagy^37^. As expected, expression of *Park2* alone had little effect on the fluorescence of mitochondria-localized GFP whereas further addition of CCCP reduced the GFP fluorescence by 56% (Fig. 2b,c). In the presence of *Park2* and CCCP, the red patches in cytoplasm were reduced noticeably in the ePD images (Fig. 2b) and the PD index also decreased by 15% (Fig. 2c). These results indicate that the reduction of the mitochondrial content was reflected, at least in part, in the ePD image and PD index. By contrast, *Park2* and CCCP increased the fluorescence of ER-localized GFP by 2-fold despite a 34% decrease in the PD index (Fig. 2d,e), which indicates poor correlation between the ER content and the PD index. Thus, although the red patches in an ePD image may derive in part from the ER, the cellular mitochondrial content is clearly reflected in the ePD image and PD index.

### PD imaging of iPSCs reveals different degrees of somatic cell reprogramming

Because the ePD image and PD index of cells reflect the mitochondrial content, the PD imaging system may be utilized for assessing changes in the mitochondrial content that occur during metabolic transitions^11^. One such example is conversions between PSCs and somatic cells; namely, differentiation of PSCs into somatic cells as well as reprogramming of somatic cells into iPSCs, both of which are accompanied by metabolic and mitochondrial alterations^38^. Therefore, the PD imaging system may be used to gauge the degrees of somatic cell reprogramming and PSC differentiation.

**Figure 3:**
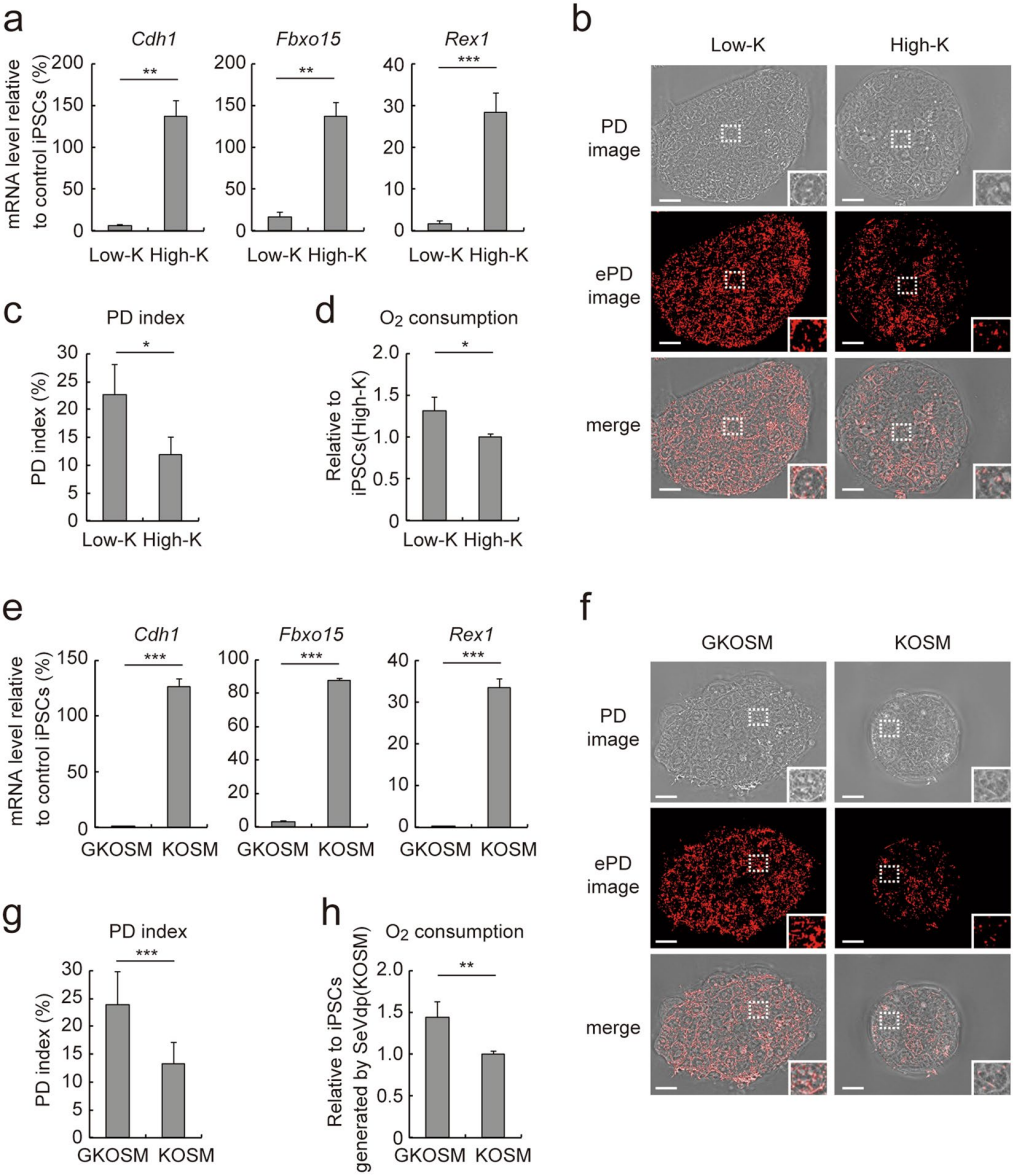
Analysis of fully- or partially-reprogrammed iPSCs by the ePD image and PD index. (**a**) *Cdh1*, *Fbxo15*, and *Rex1* mRNA levels in iPSCs(High-K) or iPSCs(Low-K) reprogrammed for 50 days from SeVdp(fK-OSM)-infected MEFs with or without 100 nM Shield1, respectively. (**b**) PD and ePD images of iPSCs(Low-K) or iPSCs(High-K) cells at day 50 of reprogramming. Selected areas are enlarged in insets at the lower right. (**c**) PD indices of the iPSCs in (**b**). (**d**) Oxygen consumption of the various iPSCs. (**e**) *Cdh1*, *Fbxo15*, and *Rex1* mRNA levels in iPSCs generated by SeVdp(GKOSM) or SeVdp(KOSM) at day 50 of reprogramming. (**f**–**h**) PD image, ePD image, PD index and oxygen consumption of iPSCs generated by SeVdp(GKOSM) or SeVdp(KOSM). Data represent means ± SEM of three independent experiments (mRNA expression and O_2_ consumption) or at least five images (PD index). *P < 0.05, **P < 0.01, ***P < 0.005. Scale bars, 10 µm.

We previously reported a stable population of partially reprogrammed iPSCs, which have stalled prematurely during iPSC generation due to a low level of KLF4 expressed from Sendai virus (SeV)-based reprogramming vectors^39^. In this system, somatic cells are reprogrammed with SeVdp(fK-OSM), which expresses destabilization domain (DD)-tagged KLF4 together with OCT4, SOX2 and c-MYC. A synthetic ligand, Shield1^40^, stabilizes DD-tagged KLF4 and therefore increases its level in cells infected with SeVdp(fK-OSM)^39^. Using this system, we generated fully reprogrammed iPSCs(High-K) and partially reprogrammed iPSCs(Low-K) by SeVdp(fK-OSM) in the presence or absence of Shield1, respectively. Conventional phase contrast microscopy failed to distinguish between iPSCs(low-K) and iPSCs(high-K) morphologically (Supplementary Fig. S4) although iPSCs(Low-K) and iPSCs(High-K) expressed markedly different levels of pluripotency markers such as *Cdh1*, *Fbxo15*, and *Rex1* (Fig. 3a) as previously reported^39^. To test if PD imaging can distinguish between them, we compared the PD images, ePD images, and PD indices of iPSCs(Low-K) and iPSCs(High-K) (Fig. 3b,c). The ePD image of iPSCs(Low-K) showed more prominent red patches than that of iPSCs(High-K) (Fig. 3b), and the PD index of iPSCs(Low-K) was larger than that of iPSCs(High-K) (Fig. 3c). Moreover, iPSCs(Low-K) consumed more oxygen than iPSCs(High-K) (Fig. 3d), consistent with a higher mitochondrial content in iPSCs(Low-K) than in iPSCs(High-K)^18^.

We also compared another set of iPSCs; partially or fully reprogrammed iPSCs generated by SeVdp(GKOSM) or SeVdp(KOSM), respectively. In the SeVdp(GKOSM) vector, which expresses GFP in addition to KLF4, OCT4, SOX2, and c-MYC, the inserted GFP gene lowers expression of the downstream KLF4 gene, as compared with the SeVdp(KOSM) vector expressing only KLF4, OCT4, SOX2, and c-MYC^39^. Figure 3e shows that iPSCs generated by SeVdp(GKOSM) expressed lower levels of *Cdh1*, *Fbxo15*, and *Rex1* than those generated by SeVdp(KOSM) as previously reported^39^. In the ePD images, the iPSCs generated by SeVdp(GKOSM) displayed more prominent red patches than those generated by SeVdp(KOSM) (Fig. 3f). The PD indices of iPSCs generated by SeVdp(GKOSM) and SeVdp(KOSM) were 22.73 ± 5.42 and 11.84 ± 3.17, respectively (Fig. 3g), consistent with higher oxygen consumption in cells generated by SeVdp(GKOSM) than by SeVdp(KOSM) (Fig. 3h).

Because iPSCs(Low-K) and iPSCs(High-K) were generated by different levels of Shield1^39^, we wished to eliminate the artifactual effect of Shield1 on PD imaging. Addition of 100 nM Shield1 to iPSCs generated by SeVdp(GKOSM) had no apparent effect on the PD image, ePD image, or PD index (Supplementary Fig. S5), indicating that Shield1 does not affect PD imaging. Taken together, these results indicate that the PD imaging system distinguishes the degree of somatic cell reprogramming by visualizing the organelles, plausibly mitochondria, in a quantifiable manner.

### PD imaging of ESCs reveals distinct degrees of ESC differentiation

**Figure 4:**
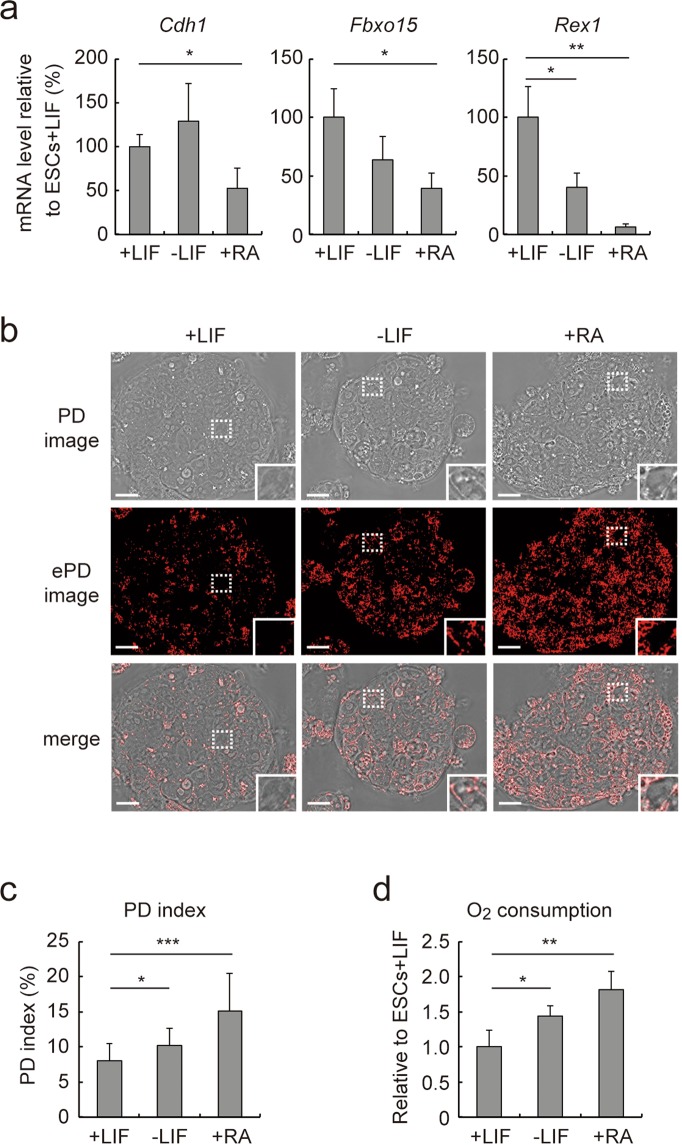
Analysis of ESC differentiation by the ePD image and PD index. (**a**) *Cdh1*, *Fbxo15*, and *Rex1* mRNA levels in ESCs cultured with LIF (+LIF), without LIF (−LIF) or with 1 µM RA (+RA) for 7 days. (**b**) PD and ePD images of differentiating ESCs. The images were acquired after 7 days of cell culture under the indicated condition. (**c**) PD indices of ESCs shown in (**b**). (**d**) Oxygen consumption of ESCs under different culture conditions. Data represent means ± SEM of three independent experiments (mRNA expression and O_2_ consumption) or at least five images (PD index). *P < 0.05, **P < 0.01, ***P < 0.005. Scale bars, 10 µm.

To test if the PD imaging system is also usable for estimating the loss of pluripotency when PSCs initiate differentiation, we cultured ESCs under conditions that promote differentiation^41^. ESCs cultured in normal serum-based medium with leukemia inhibitory factor (LIF) retained the pluripotent state as indicated by high expression levels of *Cdh1*, *Fbxo15*, and *Rex1* (Fig. 4a, +*LIF*). Withdrawal of LIF (Fig. 4a, *−LIF*) allowed ESCs to differentiate, and addition of retinoic acid (RA) in the absence of LIF (Fig. 4a, +*RA*) further promoted ESCs to undergo differentiation, as reflected by reduced expression levels of *Cdh1*, *Fbxo15*, and *Rex1*. The ePD images of ESC colonies showed that the number and intensity of red patches were lowest under the LIF(+) condition (Fig. 4b, +*LIF*), intermediate under the LIF(−) condition (Fig. 4b, *−LIF*), and highest under RA(+) condition (Fig. 4b, +*RA*). Thus, as ESCs differentiate, they increased the number and intensity of red patches. Consistent with this, the average PD indices obtained from more than ten colonies of ESCs under the LIF(+), LIF(−), and RA(+) conditions were 8.08 ± 2.40, 10.21 ± 2.45, and 15.19 ± 5.32, respectively (Fig. 4c). Moreover, the relative levels of oxygen consumption of ESCs under the LIF(+), LIF(−), and RA(+) conditions were 1.00 ± 0.24, 1.44 ± 0.14, and 1.83 ± 0.25, respectively (Fig. 4d), showing the PD index and oxygen consumption increase simultaneously during ESC differentiation. These results indicate that the PD imaging system can be utilized to monitor progression of differentiation and loss of pluripotency in cultured ESCs. Interestingly, upon initiation of differentiation, ESCs in the periphery of a colony displayed more prominent red patches than those in the core (Fig. 4b), in agreement with the greater propensity of ESCs in the periphery of a colony to move toward differentiation^42^.

### The PD index permits unbiased selection of iPSC colonies with high pluripotency

**Figure 5:**
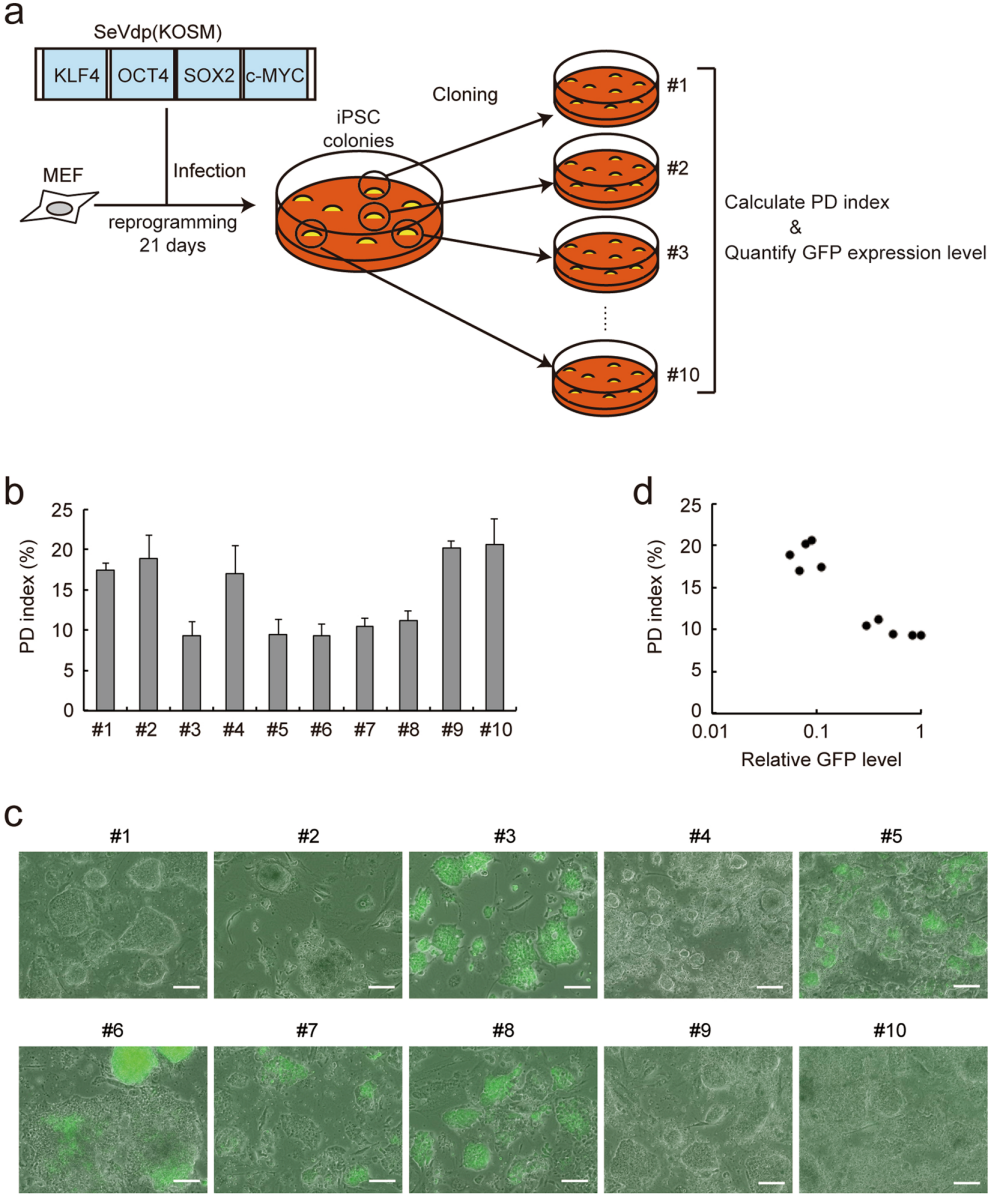
Selection of iPSCs with high pluripotency based upon the PD index. (**a**) Scheme of isolation and data collection of iPSC clones. MEFs harboring the GFP reporter gene on an inactive X chromosome were reprogrammed by the SeVdp(KOSM) vector, and colonies were cloned randomly at day 21. (**b**) PD indices of isolated iPSC clones. Data represent means ± SEM of the PD indices obtained from at least five images. (**c**) GFP fluorescence in isolated iPSC clones described in (**b**). GFP fluorescence of each iPSC clone was observed by phase contrast fluorescence microscopy. Scale bars, 100 µm. (**d**) Relationship between the GFP fluorescence and PD index. The intensity of GFP fluorescence in each iPSC clone was quantified using AxioVision software.

To validate the usability of the PD index for selecting iPSC colonies with high pluripotency, we used SeVdp(KOSM) to generate iPSCs from female mouse embryonic fibroblasts (MEFs) that carry the transgenic GFP gene on an X chromosome^43^. Because the GFP gene is randomly inactivated in the MEFs, fluorescence activated cell sorting (FACS) was performed to isolate only GFP-negative MEFs that carry the GFP transgene on an inactive X chromosome, prior to reprogramming. When fully reprogrammed into iPSCs, the GFP-negative MEFs are expected to initiate GFP expression upon X chromosome reactivation, a late event during reprogramming that is indicative of high pluripotency^44^. Twenty-one days after reprogramming, ten colonies of iPSCs generated from the MEFs harboring the GFP reporter gene on an inactive X chromosome were picked up at random, and each colony was divided in half and passaged into corresponding wells in two separate plates (Fig. 5a). One plate was analyzed first by the PD imaging system and then another by a phase contrast fluorescence microscope independently (Fig. 5a). The PD indices from ePD images showed two distinct types of clones; those with the PD index approximately above 15 (clones #1, #2, #4, #9, and #10) or below 15 (clones #3, #5, #6, #7, and #8) (Fig. 5b). The fluorescence microscopy showed that GFP was expressed clearly in clones #3, #5, #6, #7, and #8, whereas other clones showed very low GFP expression (Fig. 5c), indicating that clones #3, #5, #6, #7, and #8 have undergone X chromosome reactivation and are more advanced in reprogramming than other clones. When the PD indices and GFP expression levels were plotted on a single graph, these iPSC clones were segregated into two distinct groups (Fig. 5d). The cells with the PD index below 15 expressed high levels of GFP, whereas those with the PD index above 15 showed virtually no GFP expression, demonstrating an inverse relationship between the PD index and GFP expression. Because GFP expression indicates X chromosome reactivation in the female MEFs harboring the GFP reporter gene on an inactive X chromosome and thus high pluripotency, the PD index may serve as a usable indicator for evaluating the pluripotency of live PSC colonies.

### 3D structure of PSC colonies produced by PD imaging serves as an indicator of pluripotency

**Figure 6:**
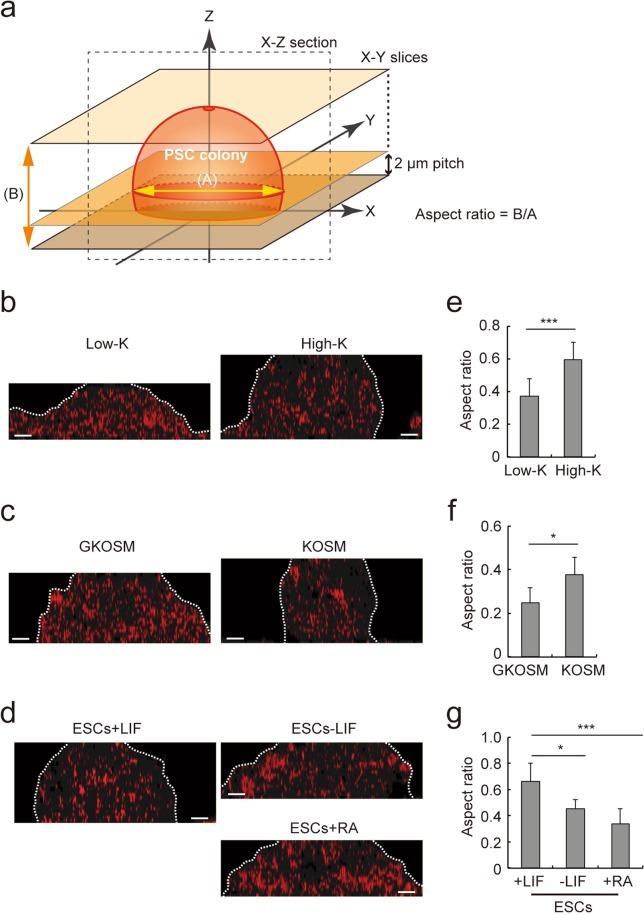
3D morphological analysis of PSC colonies by ePD images. (**a**) Outline of reconstituting a 3D ePD image of a PSC colony and derivation of its aspect ratio. A serial z-stack ePD images (X-Y slices) were combined to reconstitute a 3D ePD image of a PSC colony. The 3D ePD image was used to produce a vertical section (X-Z section) of the colony. The aspect ratio of a colony was calculated by dividing its height (B) by diameter (A). The diameter (A) of a colony was defined as the diameter of an imaginary circle, the area of which is identical to that of the maximum cross section of the colony. (**b**–**d**) Vertical X-Z sections of the 3D ePD images of PSC colonies. The 3D ePD images, reconstituted by combining the z-stack ePD images, were used to produce cross sections of the colonies for iPSCs(Low-K) and iPSCs(High-K) (**b**), iPSCs generated by SeVdp(GKOSM) and SeVdp(KOSM) (**c**), or ESCs cultured as in Fig. 4 (**d**). The contour of each colony is indicated by dotted lines. Scale bars, 10 µm. (**e**–**g**) Aspect ratios of colonies in each set of PSCs. The aspect ratios of partially- or fully-reprogrammed iPSCs (**e**,**f**) or ESCs with distinct degrees of differentiation (**g**) were calculated as indicated in (**a**). Data represent means ± SEM of aspect ratios of at least five colonies. *P < 0.05, ***P < 0.005. Scale bars, 10 µm.

A 3D image of cells is typically reconstructed by multiple fluorescent images obtained by confocal microscopy, which normally requires labeling of the cells. Even without any labeling, however, the PD imaging system produces a series of ePD images of a PSC colony along the z-axis, which can be overlaid to reconstruct a 3D image of the colony (Figs 1d and 6a and Supplementary Videos S1, S2). Because mouse PSCs have a tendency to form dome-like colonies in cell culture, we compared the 3D ePD images of partially or fully reprogrammed iPSCs (Fig. 6). The red patches in a vertical section of the 3D ePD images were more prominent in PSCs with lower pluripotency (Fig. 6b–d). In addition, vertical sections of reconstructed 3D ePD images of iPSCs(Low-K) and iPSCs(High-K) colonies showed clearly different shapes, with the colony of iPSCs(High-K) vertically longer and more spherical than that of iPSCs(Low-K) (Fig. 6b). Similarly, iPSCs generated by SeVdp(KOSM) formed a high and spherical colony as compared with those generated by SeVdp(GKOSM) (Fig. 6c and Supplementary Videos S1, S2), indicating that iPSC colonies adopt a vertically longer and rounder structure when more advanced in reprogramming. We also analyzed the colonies of ESCs that were induced toward differentiation. As shown in Fig. 6d, ESCs cultured with LIF formed a dome-like colony, which became flatter when ESCs were allowed to differentiate in the absence of LIF or by further addition of RA. These results indicate that the 3D ePD image clearly captures morphological changes of PSC colonies during reprogramming and differentiation without any labeling of cells.

To quantitatively express the roundness of PSC colonies, we next calculated the aspect ratio of each colony from 3D ePD images. The aspect ratio is defined as the height divided by the diameter and was calculated by measuring the height and maximum diameter of a colony in the 3D ePD images (Fig. 6a). We picked up more than five colonies to determine the aspect ratio of each colony, and the obtained aspect ratios were averaged (Fig. 6e–g). Aspect ratios of colonies for iPSCs(Low-K) and iPSCs(High-K) were 0.375 ± 0.104 and 0.594 ± 0.108, respectively (Fig. 6e), and those for iPSCs generated by SeVdp(GKOSM) and SeVdp(KOSM) were 0.250 ± 0.067 and 0.379 ± 0.079, respectively (Fig. 6f). Thus, fully reprogrammed iPSCs formed colonies with a larger aspect ratio than partially reprogrammed iPSCs, demonstrating quantitatively that iPSC colonies adopt higher dome-like morphology as they advance in reprogramming. Furthermore, aspect ratios of ESCs with LIF, without LIF, and with RA were 0.663 ± 0.142, 0.457 ± 0.070, and 0.340 ± 0.117, respectively (Fig. 6g), showing again that ESC colonies become flatter as they differentiate. These results indicate that PD imaging of PSC colonies permits reconstruction of their 3D structures and their aspect ratios serve as an indicator of pluripotency of PSCs.

### The PD imaging system reveals the internal organization of PSC colonies

**Figure 7:**
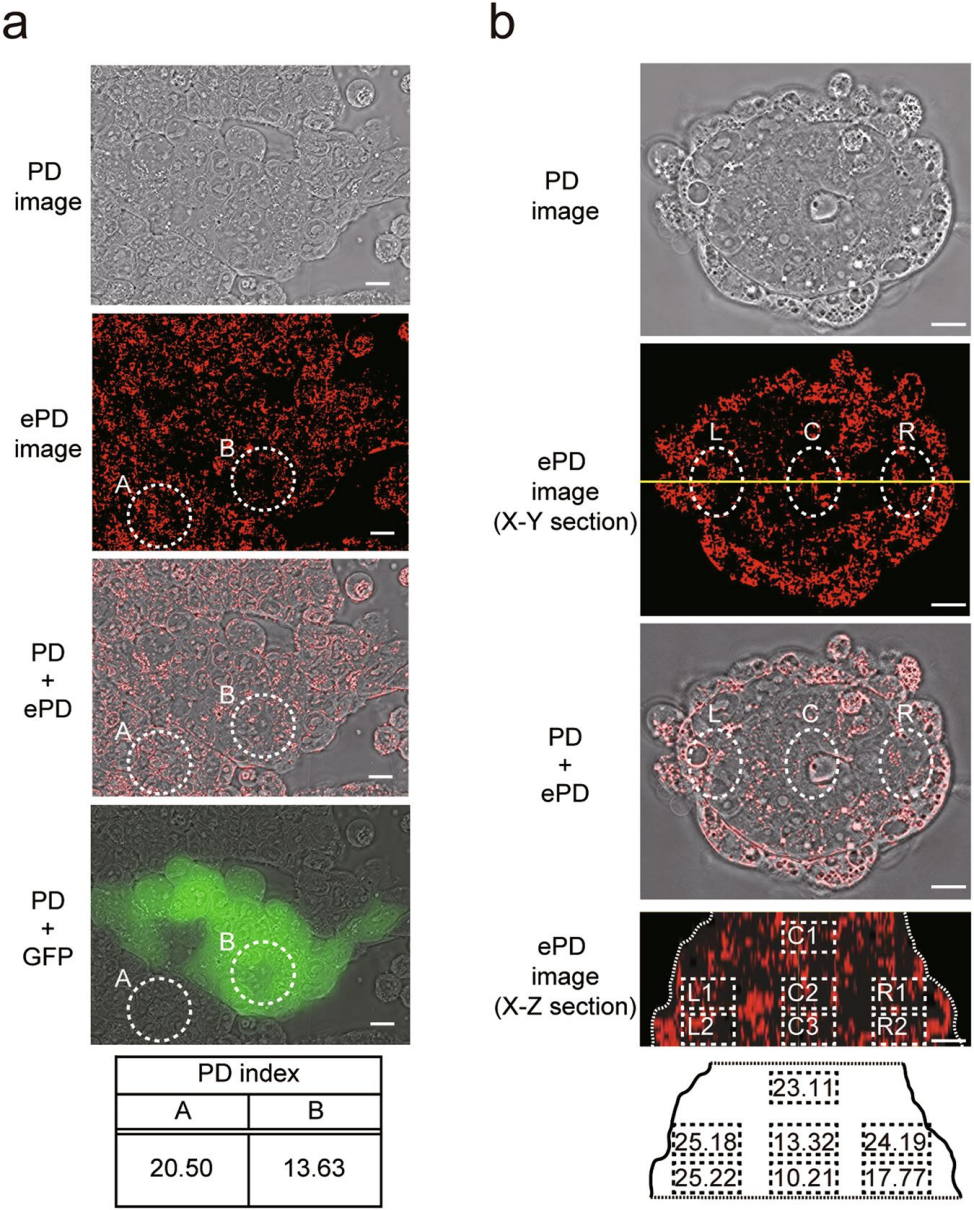
Internal heterogeneity of PSC colonies as revealed by ePD images. (**a**) Relationship between GFP fluorescence and the PD index of iPSCs in a single colony. FACS-sorted MEFs harboring the GFP reporter gene on an inactive X chromosome were reprogrammed using the SeVdp(KOSM) vector. At day 25 of reprogramming, the PD imaging system was used to observe an iPSC colony, in which only a small minority of iPSCs expressed GFP fluorescence. The PD indices of GFP(−) (A) and GFP(+) cells (B), surrounded by dotted lines, are shown in the bottom panel. Scale bars, 10 µm. (**b**) PD indices of different regions within a three-dimensionally reconstructed ESC colony, which was induced toward differentiation. ESCs were cultured with 1 µM RA and without LIF for 7 days. The yellow line in the X-Y section image indicates the position of slice through the colony to obtain the X-Z section image. PD indices of the regions encircled by dotted rectangles were measured by the PD imaging system and shown in the bottom diagram. The contour of the colony is outlined by dotted lines. Scale bars, 10 µm.

In addition to the heterogeneity of pluripotency among PSC colonies, individual PSCs within a single colony may have different degrees of pluriptency; for example, PSCs located in the periphery of a colony have a tendency to move toward differentiation^42^. Thus, using the PD imaging system, we attempted to assess the morphological heterogeneity of subcellular structures among individual cells in a single iPSC colony generated from FACS-sorted female MEFs harboring the GFP reporter gene on an inactive X chromosome (Fig. 5). We chose an iPSC colony that had GFP-positive and -negative cells within its different locations. Figure 7a shows a colony comprised of cells that have undergone X chromosome reactivation (GFP-positive) as well as those that retain an inactive X chromosome (GFP-negative) (Fig. 7a, *encircled locations A and B in the images*). We produced the ePD image of this colony to calculate the PD index of cells in each location. The GFP-positive cells showed a low PD index (13.63) whereas the GFP-negative cells showed a high PD index (20.50), indicating that the PD index reveals the different degree of pluripotency even within a single colony.

We also obtained the PD indices from 7 different locations within a 3D ePD image derived from a single ESC colony, which was cultured in the presence of RA for 7 days. As shown in Fig. 7b, the PD index was lower in the core of the colony (Fig. 7b, *C2*, *C3*) but higher in its periphery (Fig. 7b, *C1*, *L1*, *L2*, *R1*, and *R2*). The distribution of the PD indices within a colony is consistent with the relatively high mitochondrial content of peripheral cells, which have the tendency to move toward differentiation^11^. Together, these results show that the PD imaging system provides information of live cells within a single colony to reveal its internal heterogeneity.

## Discussion

Here we describe the PD imaging system that enables visualization of subcellular structures in unstained and unlabeled live cells. The system was developed from the conventional RM-DIC system and is equipped additionally with a pair of shear direction changers. The two RM-DIC images obtained from different directions are decomposed, deconvoluted, and compounded before integrating into a single image, which is detailed enough to highlight organelles including nucleus, nucleoli, and mitochondria within a live cell (Fig. 1). Among the subcellular structures visualized in PD images, we focused here on the cytoplasmic granular spots, whose appearance was initially noted to differ among various types of cells. Although the granular spots appeared to co-localize with mitochondria, their proximity to the ER^45^ prevented us from identifying the exact origin of the granular spots (Fig. 2). Regardless of their identity, however, the amount of granular spots, which was extracted as red patches in ePD images and quantified as the PD index, reflected the mitochondrial rather than ER content (Fig. 2). These results suggest that the PD imaging system provides useful biological information from live cells that undergo changes in the mitochondrial functions such as mitophagy and metabolic change.

It is known that mitochondria undergo dynamic changes in structure and function during iPSC generation due to the metabolic shift from oxidative phosphorylation to glycolysis^38^. We found that the PD imaging system distinguishes between partially and fully reprogrammed iPSCs, which have undergone the metabolic shift to different extents^18^. Moreover, when a heterogeneous mixture of iPSC colonies and isolated iPSC clones were analyzed by the PD imaging system, the PD index and aspect ratio of iPSC colonies were found to predict the quality of each iPSC colony without use of invasive methods (Figs 3, 5, 6). The PD index, which reflects cell metabolism, may also be applicable to human iPSCs because similar metabolic changes occur during human iPSC generation^14^. Thus, the PD imaging system will enable pre-selection of iPSC colonies with potentially high pluripotency and would reduce the time and cost before subsequent detailed analyses of iPSCs. Moreover, the PD imaging system could be combined with a time-lapse imaging and automatic cell culture system to construct a high throughput system for pre-selecting iPSCs with high pluripotency^46^.

Apart from granular spots in cytoplasm, PD images clearly visualized nucleoli, which were de-emphasized in this study when we produced ePD images from PD images. Given somewhat lower values of phase amounts for nucleoli than those in cytoplasm, it may be possible to set alternative thresholds to produce other types of ePD images that enhance visualization of nucleoli. Nucleoli have been recognized to play important roles not only for ribosome biogenesis and cell proliferation but also for development, cancer, and cell aging as well as for various human diseases^47^. Thus, the PD imaging system may provide mechanistic insights into these cellular processes by visualizing the nucleolar structure in a quantifiable manner.

In addition to the PD index and aspect ratio, the PD imaging system produces sectional images of a PSC colony and reveals its internal structure. This feature is somewhat similar to digital holographic microscopy, which enables quantitative 3D phase imaging of unlabeled live cells^48,49^. Thus, the PD imaging system may be utilized for assessing the 3D structure of live cells or even organoids, which consist of multiple types of cells that self-organize into an orderly structure^50^. The internal structure of an organoid is not easily amenable to visualization unless they are fixed and sliced for staining and labeling. Even without such invasive methods, however, the PD imaging system may be utilized to quantitatively observe the internal structure of live organoids, which should aid in the basic research and clinical application of organoids.

## Methods

### Production of SeVdp vectors

To prepare vector-packaging cells, 2.0 × 10^5^ BHK/T7/151 M cells^51^ in a 6-well plate were transfected with each vector cDNA (2 µg) and the expression vector plasmids for SeV proteins (NP, P/C, M, F, HN, and L) (1 µg each) using Lipofectamine LTX with Plus Reagent (Thermo Fisher) and cultured at 32 °C for 6 days. Then, the packaging cells were further transfected with the expression vector plasmids for SeV M, F, and HN proteins and cultured at 32 °C for additional 4 days to rescue the SeVdp vector, which was released into the culture supernatant. The supernatant was filtered through a 0.45 µm cellulose acetate filter and stored at −80 °C until use. Titers of the SeVdp vectors were determined by examining NIH3T3 cells infected with a diluted vector suspension by immunostaining using an anti-SeV NP antibody^52^.

### Generation and maintenance of PSCs

To generate mouse iPSCs, MEFs were infected with SeVdp(KOSM), SeVdp(GKOSM), or SeVdp(fK-OSM) vector^39^ at 32 °C for 14 h. When iPSCs were generated using female MEFs isolated from transgenic mice carrying the GFP gene on one of X chromosomes^43^, GFP(−) MEFs were sorted by MoFlo Cell Sorter (Beckman Coulter) before infection with SeVdp(KOSM). The infected cells were seeded onto mitomycin C-treated neomycin resistant SNL76/7 feeder cells and grown for 7 days in KSR medium, consisting of Knockout-DMEM (Thermo Fisher) supplemented with 15% Knockout Serum Replacement (Thermo Fisher), 2 mM GlutaMAX (Thermo Fisher), 0.1 mM Non-essential amino acids (Thermo Fisher), 55 µM 2-mercaptoethanol (Thermo Fisher), 100 U/mL penicillin-streptomycin (Wako), and 1,000 U/mL LIF (Wako). At day 8, the medium was replaced by mES medium, consisting of DMEM (nacalai tesque) supplemented with 15% fetal bovine serum (Hyclone), 0.1 mM Non-essential amino acids, 55 µM 2-mercaptoethanol, 100 U/mL penicillin-streptomycin, and 1,000 U/mL LIF. iPSCs(Low-K) or iPSCs(High-K) were generated by infection with SeVdp(fK-OSM), followed by cell culturing without or with 100 nM Shield1 (Takara Bio), respectively. The intensity of the GFP fluorescence in iPSCs generated from the MEFs harboring the GFP reporter gene was determined using AxioVision software (Zeiss).

EB5 mouse ESCs (RIKEN BioResource Center) were maintained in mES medium for self-renewal. To induce differentiation, EB5 cells were cultured in either mES medium without LIF or DMEM medium with 1 µM RA (Wako) for 7 days before analyses.

### Fluorescent labeling of cells

We fused the mitochondrial localization signal peptide of mCOX8H (MPRLPPILRLLQAPAKFTVVPKAH)^34^ to the N terminus of GFP by PCR-based methods, and the GFP gene with the mitochondria localization signal peptide was inserted into the retroviral vector plasmid, pMCs∆YY1-IRES-Puro^18^. GFP fused with an ER localization signal peptide or a Golgi apparatus localization signal peptide was subcloned from ERmoxGFP^35^ or EGFP-GalT^36^ plasmid (Addgene), respectively, into pMCs∆YY1-IRES-Puro. The cDNA encoding a PARKIN protein (*Park2*) was amplified from mouse ESC cDNA and inserted into a retroviral vector plasmid, pMCs∆YY1-IRES-Neo^18^. To prepare a retrovirus stock, PLAT-E cells were transfected with each plasmid using Lipofectamine LTX with Plus Reagent. Viral supernatant was collected 2 days after transfection and filtered through a 0.45 µm cellulose acetate filter and stored at −80 °C until use. Titers of the derived retroviral vectors were determined by counting the number of puromycin- or G418-resistant NIH3T3 cells infected with a diluted vector suspension.

NIH3T3 mouse fibroblasts were cultured in DMEM medium (nacalai tesque), supplemented with 10% fetal bovine serum and 100 U/mL penicillin-streptomycin. For retroviral infection, cells were cultured with retrovirus for 2 days with 8 µg/mL hexadimethrine bromide (polybrene; Sigma) followed by selection with 2 µg/mL puromycin (nacalai tesque) or 0.5 mg/mL G418 (nacalai tesque) for 7 days. We clonally isolated puromycin-resistant cells that displayed a strong and uniform fluorescent signal in the organelles of interest.

### Acquisition of PD images

RM-DIC images were obtained by an RM-DIC microscope^30,31^, which is a DIC microscope equipped with a modulating retardation unit in the illuminating optics of a conventional Olympus IX81 microscope (Objective lens UPLSAPO 60xW (NA 1.2) [OLYMPUS], TV-lens U-TV1x [OLYMPUS], Condenser lens IX2-LWUCDA (NA0.55) [OLYMPUS], 1^st^ Nomarski Prism U-DICT [OLYMPUS] and 2^nd^ Nomarski Prism IX-DIC60 [OLYMPUS], Halogen lamp with interference filter IF550 (550 ± 30 nm) [OLYMPUS] as a light source, CCD BM-141GE (1392(h)x1040(v), pixel size 6.45 × 6.45 μm) [JAI]). The retardation of the microscope is controlled by a phase modulator, which can be governed to within ±1° accuracy by a notebook PC.

The PD imaging system is composed of the above-mentioned RM-DIC system and a pair of shear direction changers. The shear direction changers are controlled with a notebook PC (Fig. 1a) and allow acquisition of two RM-DIC images with orthogonal shear directions. The two 2^nd^ Nomarski prisms (IX-DIC60), set orthogonal to each other, are rotated by switching the position of the turret (Condenser lens IX2-LWUCDA) that holds the 2^nd^ prisms. The 1^st^ Nomarski prism (U-DICT) is rotated by the same angle as the 2^nd^ prisms mechanically via the PC, which monitors the prism’s rotation through an encoder. The mechanical rotation of the prisms requires ~3 seconds to acquire 6 images. The process of computation to produce a PD image from the two RM-DIC images is outlined in Supplementary Fig. S2^33^. First, each of the two RM-DIC images (①, ①’) was decomposed into three image components; background component with the lowest spatial frequency (②, ②’), refraction component formed by a light refracted inside the sample (③, ③’), and structure component with the highest frequency formed by a light diffracted by the structure inside the sample (④, ④’). The component with the lowest spatial frequency (②, ②’) represents background due to the unevenness of the field of view and therefore was excluded from further computation. Second, the refraction components (③, ③’) and structure components (④, ④’), which correspond to the entire cell shape and the cell microstructures, respectively, were subjected to deconvolution with the OTF (Optical Transfer Function) to compute refraction phases (⑤, ⑤’) and structure phases (⑥, ⑥’), respectively^31^. Third, the refraction phases from two shear directions (⑤, ⑤’) were compounded to compute compound refraction phase (⑦). Similarly, the structure phases from the two shear directions (⑥, ⑥’) were compounded to compute compound structure phase (⑧). Fourth, the compound refraction phase (⑦) and compound structure phase (⑧), both of which are without shear direction, were recomposed to produce a PD image (⑨).

The mathematical description that corresponds to steps 1 through 5 in the Supplementary Fig. S2 is provided as Supplementary Note.

### Production of an ePD image and calculation of the PD index

An ePD image was produced from the compound structure phase (Supplementary Fig. S2, ⑧), which was derived from two RM-DIC images with two orthogonal shear directions (Supplementary Fig. S2, ① & ①’). The middle range of the compound structure phase was extracted by setting upper and lower thresholds of the phase amount. To determine the thresholds, fifty pixels of cytoplasmic granular spots or nucleolar regions were chosen from a compound structure phase image of NIH3T3 cells, and their phase amount was determined. Because the maximum phase amount in the nucleolar regions was ~0.05 λ, the lower threshold was set at 0.05 λ (Supplementary Fig. S3a). The upper threshold was set at a triple value of 0.05 λ (i.e. 0.15 λ), which excluded outlier phase amounts in granular dots probably caused by lipid droplet (Supplementary Fig. S3a) and the phase amounts in intensely white regions probably caused by apoptotic cells (Supplementary Fig. S3c). Once determined, the same thresholds were used throughout this study for extracting compound structure phases from all the images. The extracted compound structure phase was converted into a binary image and displayed in red-based pseudo-color to produce an ePD image.

To derive the PD index, 1 to 5 slices of z-stack PD images with a 2-µm pitch were acquired for NIH3T3 cells and about 30 slices were acquired for a PSC colony from top to bottom. For each image, the PD index was obtained by dividing the number of red pixels by the total number of pixels. For NIH3T3 cells, which were covered by only a few z-stack images, the PD index was derived from the largest PD index among all the z-stack ePD images. For a PSC colony, its PD index was derived from the averaged PD indices of ten z-stack ePD images (20 µm) from the bottom of the colony. The PD indices of the z-stack images (i.e. ~20 images) above the ten images were not used for this calculation because phase amounts near the top of a colony became diminished by light scattering inside the colony.

To produce a 3D image of a colony, all the z-stack ePD images of a colony were subjected to a correction method using an attenuation coefficient of Lambert-Beer’s law because the reduction of phase amount by light scattering from cells follows this law^53^. Then, the corrected z-stack images were combined into a single 3D image by Imaris image analysis software (BITPLANE). To derive the aspect ratio, the maximum horizontal area of a colony was determined using the reconstructed 3D ePD image. The diameter of an imaginary circle, the area of which is identical to this maximum horizontal area, was defined as the diameter of the colony. The height of the colony, on the other hand, was calculated from the number of z-stack images (2 µm pitch) from bottom to top of the colony. Then, the height was divided by the diameter to derive the aspect ratio of the colony.

### Quantitative RT-PCR

To extract RNA from PSCs, they were grown on feeder cells and then passaged under a feeder-free condition to avoid any contamination of feeder cells. Total RNA was extracted using ISOGEN (Nippon Gene), and reverse transcription was performed using Superscript III First-Strand Synthesis System (Thermo Fisher). Quantitative PCR (qPCR) analyses were performed using 7500 Fast Real-time PCR System (Applied Biosystems) with GoTaq qPCR Master Mix (Promega). The expression levels were normalized against that of TATA-box binding protein (TBP). The cDNA from a mouse iPSC line that is capable of contributing to mouse chimeras^51^ or EB5 ESCs cultured with LIF were used as controls for pluripotency marker expression. The DNA sequences of the primers used for qPCR were described previously^39^.

### Determination of O_2_ consumption

1.0 × 10^5^ cells were cultured in a 96-well plate with feeder cells for one day, and the oxygen consumption was determined using Extracellular O_2_ Consumption Assay Assay Kit (Abcam) according to manufacturer’s procedures.

### Statistical analysis

Two-tailed Student’s t-tests were used to test for a statistically significant difference between data sets. A value of *P* < 0.05 was considered statistically significant.

## Supplementary information

Supplementary Information

Supplementary Movie 1

Supplementary Movie 2
