# Supplementary material for: Live-cell imaging of subcellular structures for quantitative evaluation of pluripotent stem cells
Source: Sci Rep. 2019 Feb 11;9:1777. doi: 10.1038/s41598-018-37779-x (PMC6370783; doi:10.1038/s41598-018-37779-x)
Supplement: Supplementary file 1 — Supplementary Information [file 41598_2018_37779_MOESM1_ESM.pdf]

Supplementary Information

Live-cell imaging of subcellular structures for quantitative evaluation of pluripotent stem cells

Ken Nishimura[1,4*], Hiroshi Ishiwata[2,4], Yuta Sakuragi[1], Yohei Hayashi[3], Aya Fukuda[1], Koji Hisatake[1*]

[1] Laboratory of Gene Regulation, Faculty of Medicine, University of Tsukuba, 1-1-1 Tennodai, Tsukuba, Ibaraki 305-8575, Japan.

[2] Optical Technology R&D Department 2, Optical System Development Division, Olympus Corporation, 67-4 Takakura-machi, Hachioji, Tokyo 192-0033, Japan.

[3] iPS Cell Advanced Characterization and Development Team, Bioresource Research Center, RIKEN, 3-1-1 Koyadai, Tsukuba, Ibaraki 305-0074, Japan.

[4] Co-first authors

*Correspondence should be addressed to K.N. (ken-nishimura@md.tsukuba.ac.jp) or K.H. (kojihisa@md.tsukuba.ac.jp)

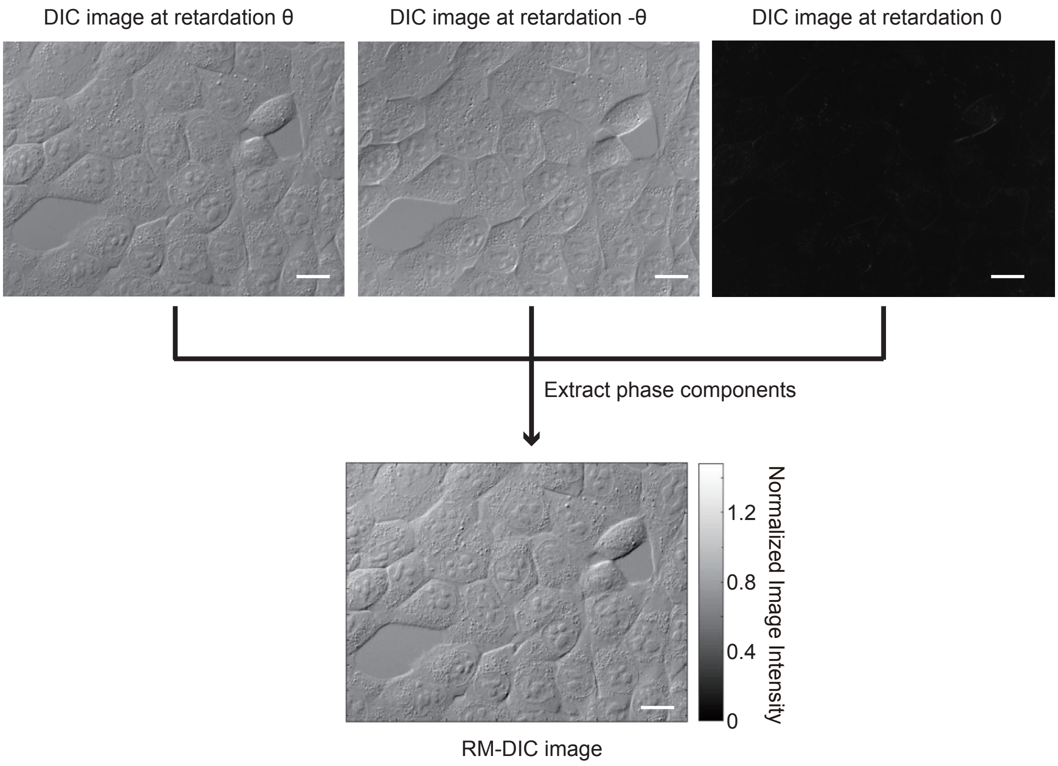

Supplementary Figure S1 Production of an RM-DIC image. DIC images with three different retardations (±θ, 0) were used to calculate phase components, which were then normalized and displayed as a 2D image termed an RM-DIC image[30,31]. In this image, the gray-scale value of each pixel represents the intensity of its phase component. Scale bars, 10 μm.

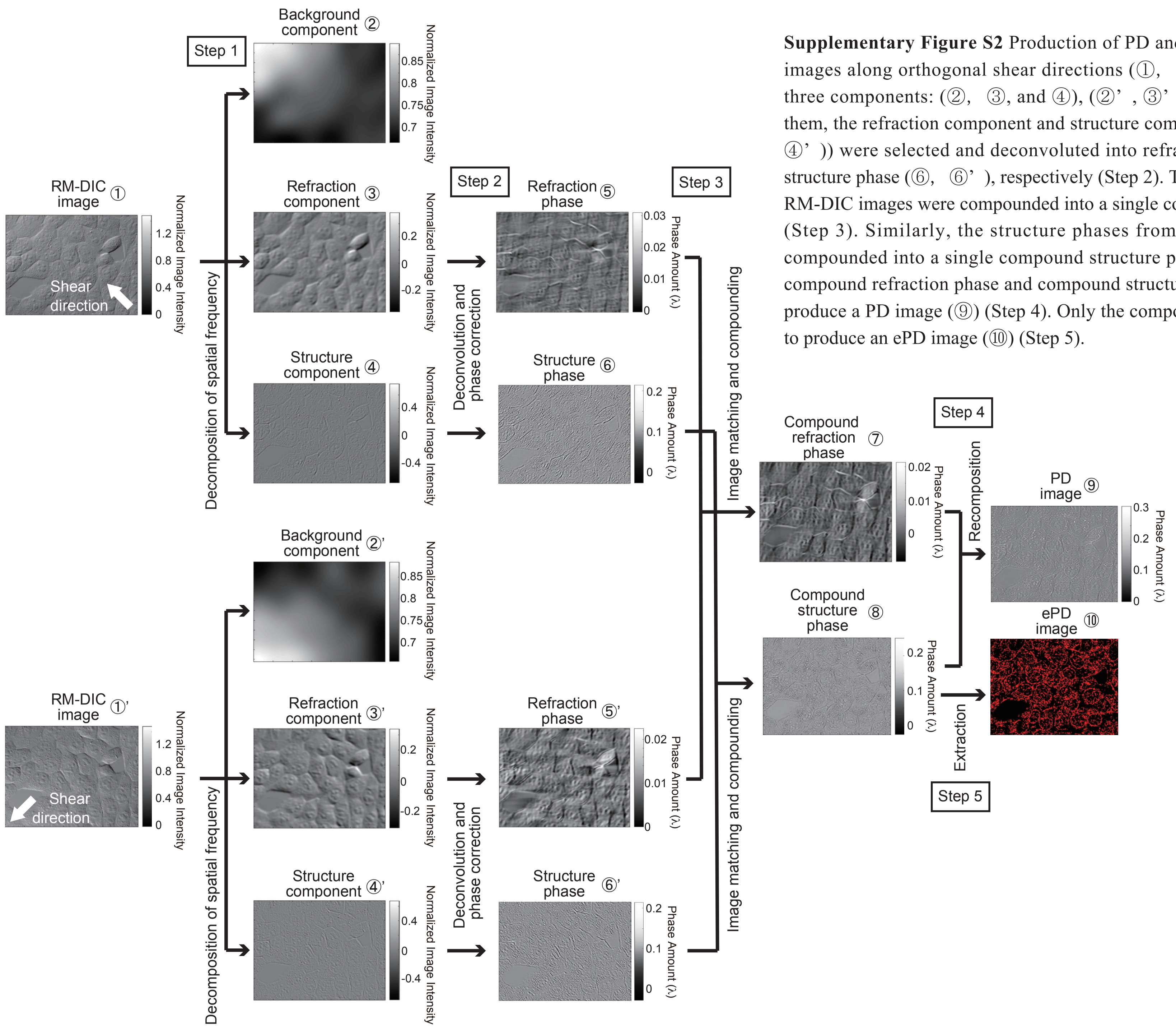

Supplementary Figure S2 Production of PD and ePD images. Two RM-DIC images along orthogonal shear directions (①, ①') were decomposed into three components: (②, ③, and ④), (②' , ③' , and ④') (Step 1). Among them, the refraction component and structure component ((③ and ④), (③' and ④')) were selected and deconvoluted into refraction phase (⑤, ⑤') and structure phase (⑥, ⑥'), respectively (Step 2). The refraction phases from two RM-DIC images were compounded into a single compound refraction phase (⑦) (Step 3). Similarly, the structure phases from two RM-DIC images were compounded into a single compound structure phase (⑧) (Step 3). Then, the compound refraction phase and compound structure phase were recomposed to produce a PD image (⑨) (Step 4). Only the compound structure phase was used to produce an ePD image (⑩) (Step 5).

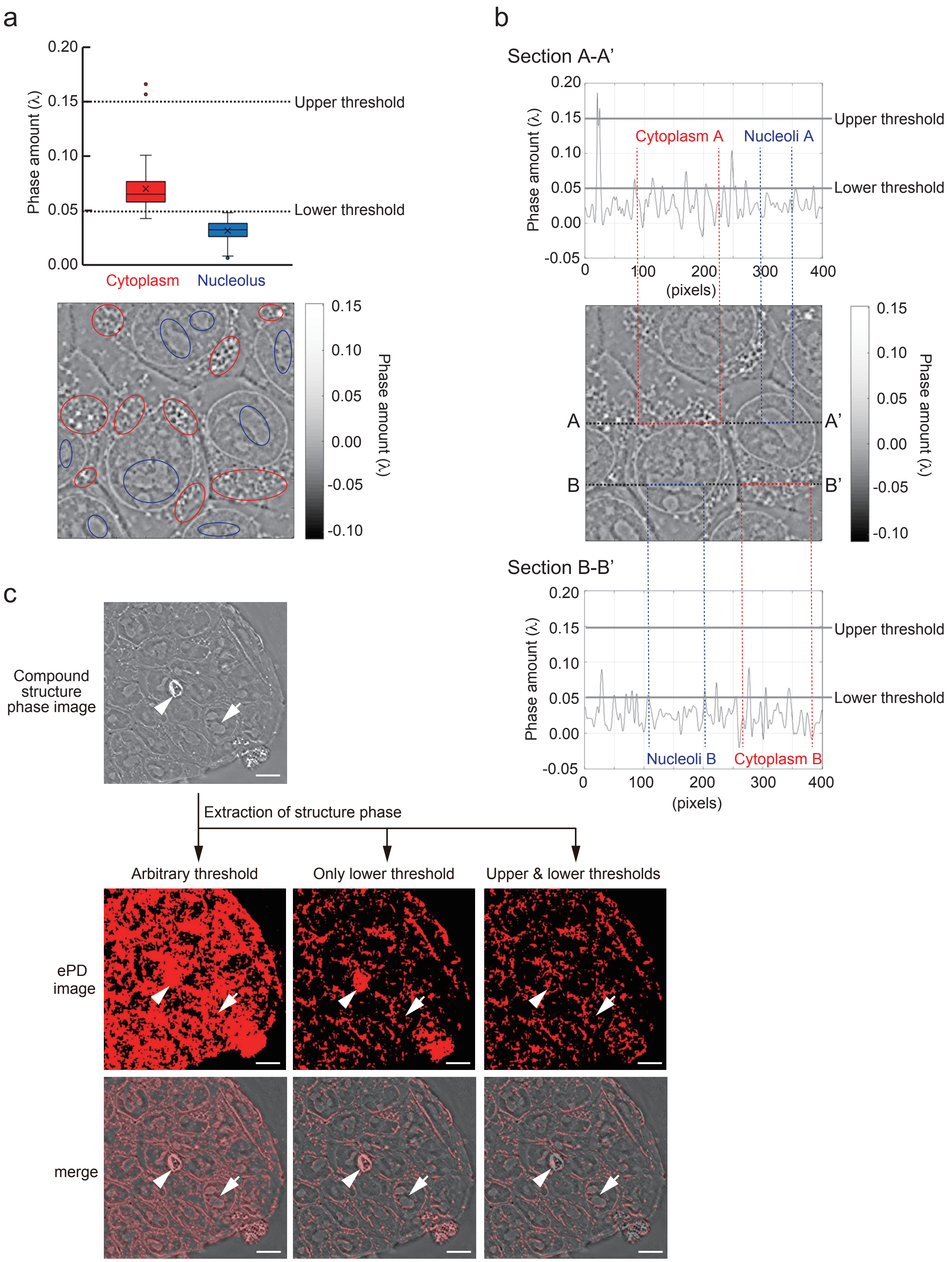

Supplementary Figure S3 Determination of thresholds and production of ePD images. **(a)** Box plot of phase amounts in fifty pixels of cytoplasmic and nucleolar regions in a compound structure phase image of NIH3T3 cells. The lower threshold (0.05 λ) used for producing ePD images was determined as the maximum phase amount of nucleolar regions. The upper threshold was set at 3x lower threshold (0.15 λ), which included most of phase amounts in cytoplasmic regions except for some outliers. The cytoplasmic (red) and nucleolar (blue) regions are encircled in the bottom image. **(b)** Phase amounts along cross sections. Phase amount along the two dotted lines (A-A' and B-B') are plotted in graphs above and below the image. Cytoplasmic or nucleolar regions are highlighted by red or blue dotted lines. **(c)** ePD images extracted from the compound structure phase image using (a) different threshold(s). Binary images produced from extracted compound structure phase with an arbitrary threshold (> 0.02 λ) (left panels), with only lower threshold (> 0.05 λ) (middle panels), and with both upper and lower thresholds (between 0.15 λ and 0.05 λ) (right panels) are shown. Note the reduction of compound structure phases of nucleoli (arrow) and dead cells (arrow head). Scale bars, 10 μm.

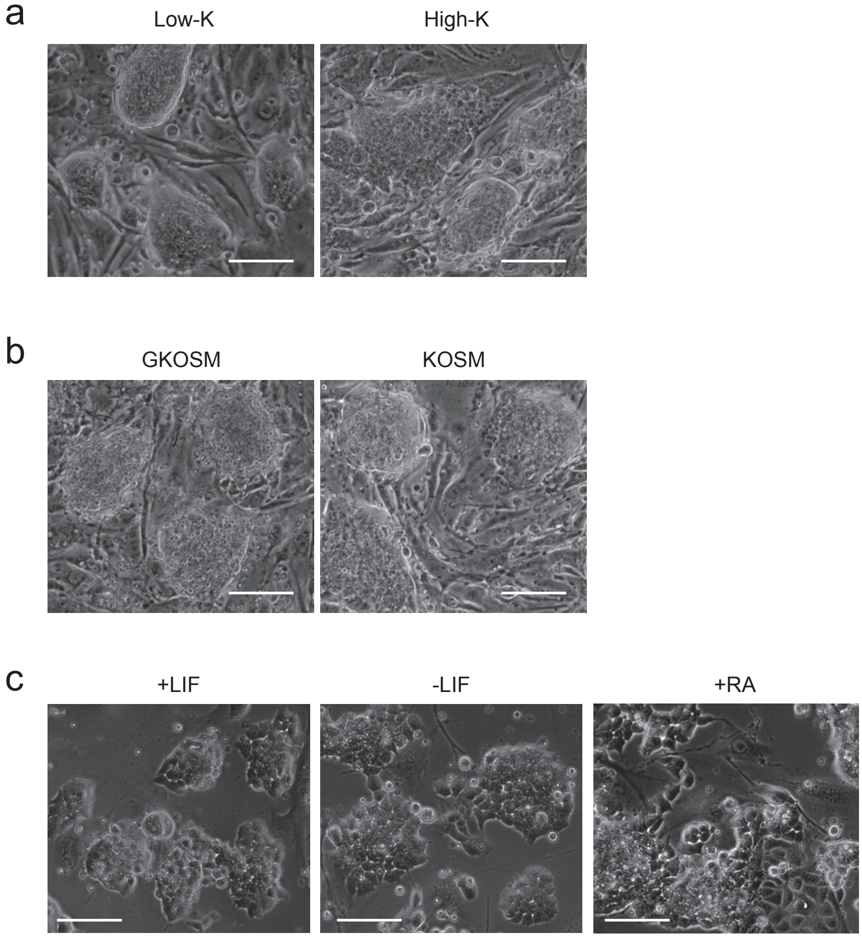

Supplementary Figure S4 Morphology of PSC colonies observed by phase contrast microscopy. Phase contrast microscopy was used to observe morphological features of iPSCs(Low-K) and iPSCs (High-K) **(a)**, iPSCs generated by SeVdp(GKOSM) and SeVdp(KOSM) **(b)**, or ESCs cultured with LIF, without LIF or with 1 μM RA for 7 days **(c)**. Scale bars, 100 μm.

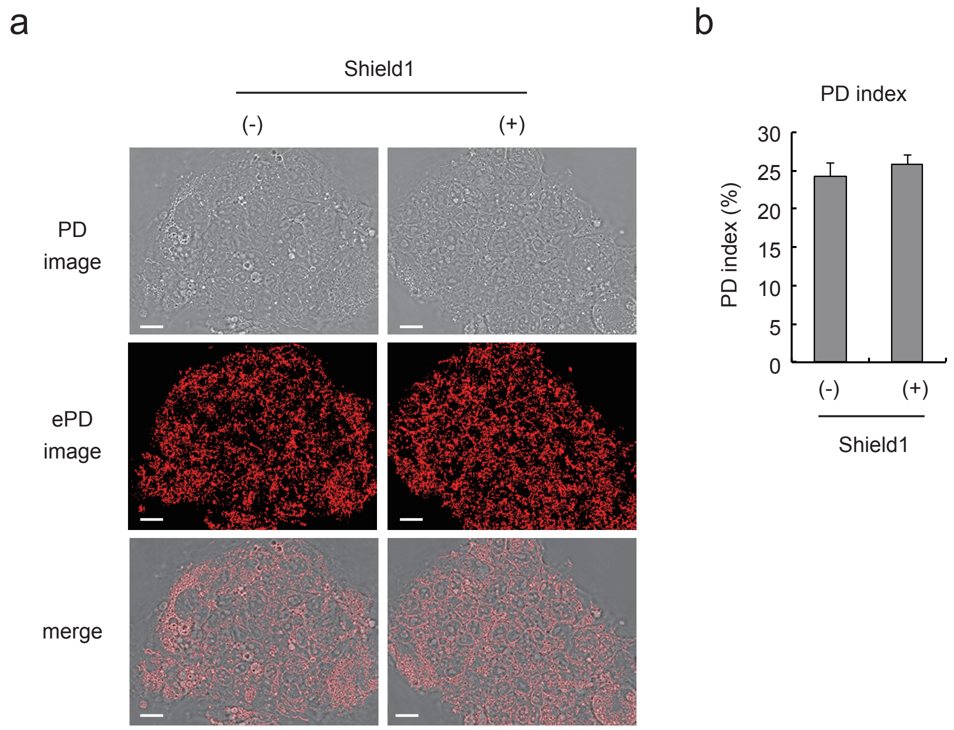

Supplementary Figure S5 Absence of the effect of Shield1 addition on the PD index. **(a)** PD and ePD images of iPSCs generated by SeVdp(GKOSM) cultured with or without 100 nM Shield1 for 14 days. **(b)** PD indices of iPSCs observed in **(a)**. Data represent means ± SEM of at least five ePD images. Scale bars, 10 μm.

Supplementary Movie 1 3D ePD image of iPSC generated by SeVdp(GKOSM). All the z-stack ePD images of a colony were combined into a single image by Imaris image analysis software.

Supplementary Movie 2 3D ePD image of iPSC generated by SeVdp(KOSM). The image was produced in essentially the same way as Supplementary Movie 1.

Supplementary Note

RM-DIC images ① and ①' are formed as below[31],

$$I_{RM-image①}(x,y,s) = \frac{I(x,y,s,\theta) - I(x,y,s,-\theta)}{I(x,y,s,\theta) + I(x,y,s,-\theta) - (cos\theta + 1)\cdot I(x,y,s,0)}$$

$$I'_{RM-image①'}(x',y',s') = \frac{I(x',y',s',\theta) - I(x',y',s',-\theta)}{I(x',y',s',\theta) + I(x',y',s',-\theta) - (cos\theta + 1)\cdot I(x',y',s',0)}$$

where s and s' are shear directions, $\pm\theta$ and 0 are the amount of retardations , $I(x, y, s, \theta)$ is each DIC image.

Step 1 (Decomposition of spatial frequency)[33]

Each RM-DIC image is broken down into the background component with the lowest spatial frequency, the refraction component formed by a light refracted inside the cell, and the structure component with the highest spatial frequency formed by a light diffracted by the structure inside the cell.

$$I_{RM-image①}(x,y,s) = I_{BG②}(x,y,s) + I_{Re③}(x,y,s) + I_{St④}(x,y,s)$$
$$I'_{RM-image①'}(x',y',s') = I_{BG②'}(x',y',s') + I_{Re③}{}'(x',y',s') + I_{St④}{}'(x',y',s')$$

Step 2 (Deconvolution and phase correction)[31]

Refraction phase ⑤ (Phase $_{Re⑤}$) is calculated by the following processes.

Refraction phase is transformed from $I_{Re③}(x,y,s)$ by

$$\emptyset_{Re}(x,y,s) = \frac{1 - cos\theta}{2sin\theta}\cdot tan^{-1}\{Decon_{Re}[I_{Re③}(x,y,s)]\}$$

Non-linearity between image intensity and phase distribution is corrected by

$$\text{Phase}_{Re⑤}(x,y,s) = \tan^{-1}\left[\frac{\emptyset_{Re}(x,y,s)}{1 - \emptyset_{Re}(x,y,s)^2/2}\right]$$

Refraction phase ⑤' (Phase $_{Re⑤'}$) shear direction s' is calculated in the same way by

$$\emptyset'_{Re}(x',y',s') = \frac{1 - cos\theta}{2sin\theta}\cdot tan^{-1}\{Decon_{Re}[I'_{Re③'}(x',y',s')]\}$$

$$\text{Phase}'_{Re⑤'}(x',y',s') = \tan^{-1}\left[\frac{\emptyset'_{Re}(x',y',s')}{1 - \emptyset'_{Re}(x',y',s')^2/2}\right]$$

Structure phase ⑥ (Phase $_{St⑥}$) is calculated by the same process by

$$\emptyset_{ST}(x,y,s) = \frac{1 - cos\theta}{2sin\theta}\cdot tan^{-1}\{Decon_{ST}[I_{ST④}(x,y,s)]\}$$

Non-linearity between the image intensity and phase distribution is corrected by

$$\mathrm{Phase}_{ST⑥}(x,y,s) = \tan^{-1}\left[\frac{\emptyset_{ST}(x,y,s)}{1-\emptyset_{ST}(x,y,s)^2/2}\right]$$

Structure phase was calculated in the same way at shear direction *s*'

$$\emptyset'_{ST}(x',y',s') = \frac{1-cos\theta}{2sin\theta}\cdot tan^{-1}\{Decon_{ST}[I'_{ST④'}(x',y',s')]\}$$

$$\mathrm{Phase}'_{ST⑥'}(x',y',s') = \tan^{-1}\left[\frac{\emptyset'_{ST}(x',y',s')}{1-\emptyset'_{ST}(x',y',s')^2/2}\right]$$

$$\mathrm{Decon}_{comp}[g(x,y)] = \mathcal{F}^{-1}[\mathcal{F}\{g(x,y)\}/iMTF_{comp}(fx,fy)]$$

where g(x) is any function with Fourier transform, $\mathcal{F}\{g(x,y)\}$ is the Fourier transform function and $\mathcal{F}^{-1}\{g(x,y)\}$ is the inverse Fourier transform function.

Step 3 (Image matching and compounding)[54,55]

When the shear direction is changed from *s* to *s*', the two DIC images at shear directions *s* and *s*' shift relative to each other due to the wedge of Nomarski prisms.

Here the structure phases, $\mathrm{Phase}_{St⑥}$ and $\mathrm{Phase}_{St⑥'}$, were used to calculate the relative shift (m, n) by the POC (Phase Only Correlation) method.

(m, n) = POC{$\mathrm{Phase}_{St⑥}$, $\mathrm{Phase}_{St⑥'}$}

Then the image shifts of refraction phase ⑤' and structure phase ⑥' are corrected with the relative shift (m, n).

Each component is then compounded as

$$\mathrm{Phase}_{Re⑦}(x,y) = \sqrt{\mathrm{Phase}_{Re⑤}(x,y,s)^2 + \mathrm{Phase}_{Re⑤'}(x,y,s')^2}$$

$$\mathrm{Phase}_{St⑧}(x,y) = \sqrt{\mathrm{Phase}_{St⑥}(x,y,s)^2 + \mathrm{Phase}_{St⑥'}(x,y,s')^2}$$

Step 4 (PD image recomposition)[33]

$$\mathrm{PD\ image⑨}(x,y) = \mathrm{Phase}_{Re⑦}(x,y) + \mathrm{Phase}_{St⑧}(x,y)$$

Step 5 (ePD image extraction)

We set two thresholds as described in Supplementary Figure S3 and extract an ePD image

$$\mathrm{ePD\ image⑩}(x,y) = 0.05\,\lambda < \mathrm{Phase}_{St⑧}(x,y) < 0.15\,\lambda$$

References

54. Horner, J. L. & Gianino, P. D. Phase-only matched filtering. *Appl. Opt.* **23**, 812 (1984).

55. Ishiwata, H., Nagai, H., Naka, T. & Itoh, M. Widening the range of high-precision quantitative measurement in retardation-modulated differential interference microscope. *Opt. Rev.* **17**, 214-217 (2010).
